# Recent Advances in Biotransformation of Saponins

**DOI:** 10.3390/molecules24132365

**Published:** 2019-06-26

**Authors:** Yi He, Zhuoyu Hu, Aoran Li, Zhenzhou Zhu, Ning Yang, Zixuan Ying, Jingren He, Chengtao Wang, Sheng Yin, Shuiyuan Cheng

**Affiliations:** 1 National R&D Center for Se-rich Agricultural Products Processing, College of Food Science and Engineering, Wuhan Polytechnic University, Wuhan 430023, China; 2 Key Laboratory for Deep Processing of Major Grain and Oil, Ministry of Education, Wuhan 430023, China; 3 Hubei Key Laboratory for Processing and Transformation of Agricultural Products, Wuhan Polytechnic University, Wuhan 430023, China; 4 Beijing Engineering and Technology Research Center of Food Additives, Beijing Technology & Business University (BTBU), Beijing 100048, China

**Keywords:** saponins, biotransformation, microorganisms, bioavailability

## Abstract

Saponins are a class of glycosides whose aglycones can be either triterpenes or helical spirostanes. It is commonly recognized that these active ingredients are widely found in various kinds of advanced plants. Rare saponins, a special type of the saponins class, are able to enhance bidirectional immune regulation and memory, and have anti-lipid oxidation, anticancer, and antifatigue capabilities, but they are infrequent in nature. Moreover, the in vivo absorption rate of saponins is exceedingly low, which restricts their functions. Under such circumstances, the biotransformation of these ingredients from normal saponins—which are not be easily adsorbed by human bodies—is preferred nowadays. This process has multiple advantages, including strong specificity, mild conditions, and fewer byproducts. In this paper, the biotransformation of natural saponins—such as ginsenoside, gypenoside, glycyrrhizin, saikosaponin, dioscin, timosaponin, astragaloside and ardipusilloside—through microorganisms (*Aspergillus* sp., lactic acid bacteria, bacilli, and intestinal microbes) will be reviewed and prospected.

## 1. Introduction

**Figure 1:**
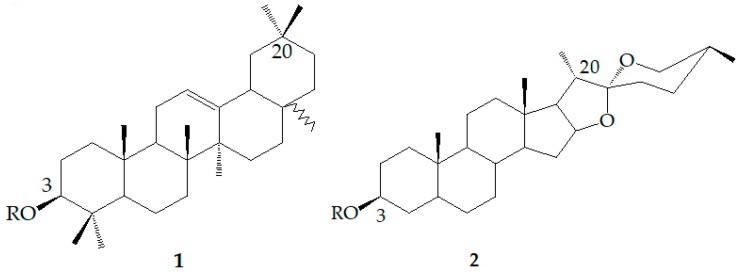
Chemical structures of triterpenoid saponin (**1**) and steroidal saponins (**2**). R = sugar moiety.

Saponins are glycosides with triterpenoids or spirostanol as glycosides, which are widely found in terrestrial higher plants and a few in marine organisms such as starfish and sea cucumbers. Saponins can be roughly divided into two categories according to their aglycone structures: triterpenoid saponins (**1**) and steroidal saponins (**2**). Both are derived from oxidized polymers containing 30 carbon atoms [1], but the difference is that triterpenoid saponins still retain 30 carbon atoms, while three methyl groups are removed from steroidal saponins (Figure 1). Saponins are considered to be the main functional components of many plant drugs and folk medicines, as well as the main components of many pharmacological properties [2]. Saponins have a major function in enhancing bidirectional immune regulation [3], reducing cholesterol [4,5], and antitumor activity [6]. Experiments have demonstrated that saponins can destroy the homeostasis of Ca^2+^ by inducing cell arrest and apoptosis as a basis for their anticancer properties. They also can protect cells from damage caused by ischemia and hypoxia [7]. However, the absorption rate of saponins in the human gastrointestinal tract is very low, but the rare saponins—of which glycosyl groups are hydrolyzed—are more easily absorbed. How to transform saponins into rare saponins is a focus of scientific research. Natural saponins can be hydrolyzed by physical (heating), chemical (acidolysis), microbial transformation, among other processes. The products include glycosides, secondary glycosides, or their derivatives. In recent years, microbial transformation has gradually become the main method to produce active secondary saponins on a large scale due to its strong specificity, mild conditions, and fewer byproducts.

The biotransformation of saponins mainly includes microbial transformation and intestinal microflora transformation. The main way of microbial transformation is through the hydrolysis of saponin glycosyl groups, so as to transform natural saponins into rare saponins containing low sugar chains. Rare saponins and their derivatives show more valuable drug effects than natural saponins. Under the action of intestinal microflora, a series of structural changes can occur and are mainly caused by the step-by-step desugar process, and the generated conversion products can have better bioavailability or stronger biological activity than the original saponins [8]. The transformation of intestinal microflora of natural saponins has attracted increasing attention. This paper reviews the research progress of natural saponin biotransformation, and provides ideas and references for the research of saponin biotransformation.

## 2. Ginsenoside

Ginseng is a perennial herbaceous plant belonging to the family Araliaceae, known as the “King of hundred herbs”. Ginseng has a wide history of clinical application in emergency treatment, cardiovascular disease, diabetes, liver, and stomach diseases. As the main active components of ginseng, 182 kinds of ginsenosides have been found to date. More than 50 kinds of ginsenosides have been isolated and identified, which are mainly divided into propanaxadiol-type saponins (PPD), propanaxtriol-type saponins (PPT), and oleanane-type saponins (oleanolic saponins) [9]. Direct absorbance of the natural ginsenosides is difficult and they first need to be transformed into secondary saponins by the metabolism of gastrointestinal flora before they can be readily absorbed and utilized in the blood, however, secondary saponins are rarely found in nature. Therefore, the biotransformation of ginsenosides into secondary saponins is a hotspot of current research.

**Figure 2:**
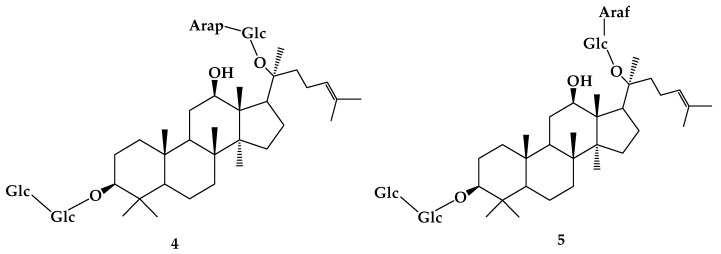
The chemical structures of compound **4**,**5**.

**Figure 3:**
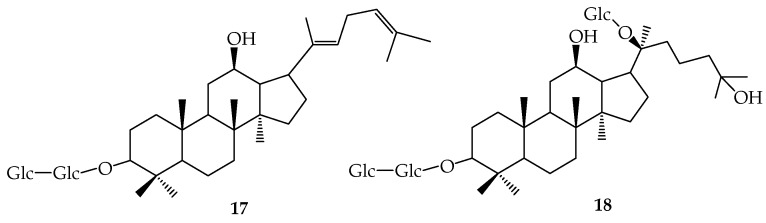
The chemical structures of compound **17**,**18**.

**Figure 4:**
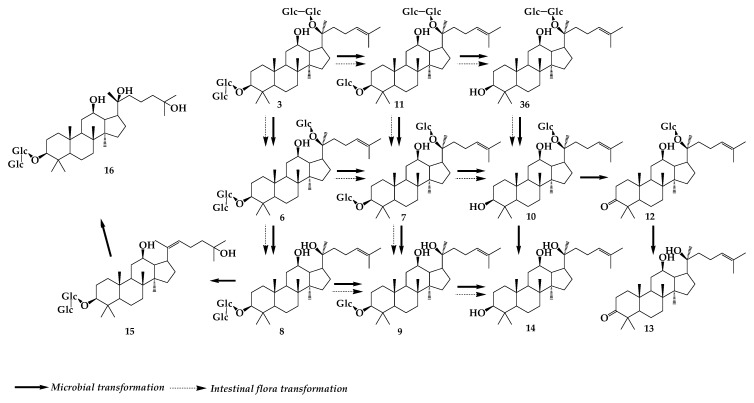
Ginsenoside transformation pathway.

Ginsenosides, including Rb1 (**3**), Rb2 (**4**), Rc (**5**) (Figure 2), and Rd (**6**), have more glycosyl groups on C-3 and C-20 than rare ginsenosides, such as F2 (**7**), Rg3 (**8**), Rh2 (**9**), and C-K (**10**). Therefore, rare ginsenosides with high activity can be prepared by deglycosylation and hydrolysis to reduce the number of glycosyl groups in ginsenosides. Studies have shown that deglycosylation and hydrolysis reactions are mainly concentrated in intestinal flora and microbial transformation, and only deglycosylation occurs in enzymatic transformation. After biotransformation of β-glucosidase produced by *Lactobacillus pentosus* strain 6105, which was isolated from kimchi, the products of **3** were identified by ^1^H NMR and ^13^C NMR, and its transformation pathway was revealed: **3**→gypenoside XVII (**11**) →**7**→**10**, **1**→**6**→**7**→**10** [10]. Quan et al. [11] discovered approximately the same transformation pathway of ginsenoside Rb1 with *Lactobacillus paralimentarius* LH4 which was isolated from kimchi. However, the ginsenoside **3** transformation pathway was different when using β-glucosidase extracted from *Microbacterium esteraromaticum* [12]. The different **3** transformation pathways under the same β-glucosidase maybe due to their diverse production strains. Using **3** as substrate, four known metabolites and two new metabolites (3-oxo-CK (**12**) and 3-oxo-PPD (**13**)) were obtained by *Cladosporium cladosporioides* fermentation. The typical fermentation route is **3**→**6**→**7**→**10**→PPD (**14**) and **12**→**13** [13]. *Aspergillus niger* strain TH-10a can effectively transform ginsenoside **3** into **6** [14]. Eight metabolites were obtained by scaled fermentation of **3** with *Acremonium strictum* AS 3.2058, including **6**, **7**, **8**, **10**, **11**, and three special compounds: **12**, 12α,25-dihydroxydammar-(E)-20-ene-3-*O*-β-d-glucopyranosyl-(1→2)-β-d-glucopyranoside (**15**) and 12β,20(R),25-trihydroxydammar-3-*O*-β-d-glucopyranosyl-(1→2)-β-d-glucopyranoside (**16**) [15]. Comparing the metabolites of **3** in rats with those in microorganisms, results revealed that **3** was transformed into ten metabolites in rats and eight of them were the same as the microbial metabolites. In addition, two new metabolites 20,24-diene-ginsenoside Rg3 (**17**) and 25-hydroxyginsenoside Rd (**18**) (Figure 3) were detected in rat feces and urine, respectively [16]. These results demonstrate that **3** has the same transformation pathway in mammalian metabolism and microbial metabolism, which demonstrates that the application of the microbial model in mammalian metabolism is reasonable. (Figure 4).

**Figure 5:**
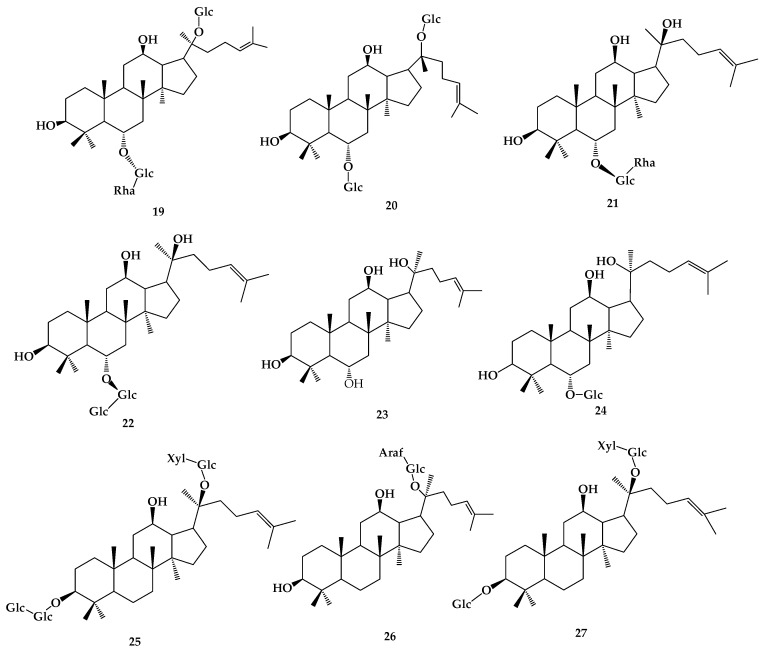
The chemical structures of compound **19**–**27**.

Ginsenoside transformation pathway Current studies on the biotransformation of ginsenosides are not limited to the transformation of Rb1. In Seong-Eun Park’s study [17], **3**, **4**, **5**, and Re (**19**) can be transformed into **6**, Rg1 (**20**) and Rg2 (**21**) by lactic acid bacteria. The conversion of **6**, **20**, and **21** into smaller deglycosylated forms was more rapid than that of **6** from **3**, **4**, and **5**, as well as that of **20** and **21** from **19** during the first 2 d of fermentation. **8** can be transformed into **9** by *Esteya vermicola* CNU 120806, and three important influencing factors including pH, temperature and substrate concentration were optimized. When the temperature is 50 °C, pH is 5.0, and substrate concentration is 3 mg/mL, the conversion of **9** can reach 90.7%. It was confirmed that this conversion was the hydrolysis on only C-3 of **8** [18]. In Liu’s study [19], rare ginsenoside Rf (**22**) was transformed into 20(S)-protopanaxatriol (PPT(S)) (**23**) by glycosidase from *Aspergillus niger*. Under the condition of pH 5.0, 55 °C, and substrate concentration of 1.25 mmol/L, the yield reached 90.4%, and the transformation route was determined by HPLC as 22→Rh1(S) (**24**) →23. Biotransformation of Rb3 (**25**), **4**, and **5** have also been reported. Compound Mc (**26**) can be transformed by *Fusarium sacchari* directly from **25**, or from **25** via Gy-IX (**27**) (Figure 5) [20]. **4** and **5** were transformed into **10** via **6** and **7** by *Bifidobacterium* sp. Int57 and *Bifidobacterium* sp. SJ32. *Lactobacillus delbrueckii* transformed **4** and **5** into ginsenoside **9**, while *Bifidobacterium* sp. SH5 transformed **4** and **5** into **7**. *Aspergillus niger* transformed **4** into **10** via compound O and compound Y, whereas it transformed **5** into **10** via compound Mc [21]. In addition, some transformation ways of main ginsenosides were reported: **3**→**6**→**7**→**10**, **4**→compound O→compound Y→**10**, **5**→compound Mc→**10** [22].

**Figure 6:**
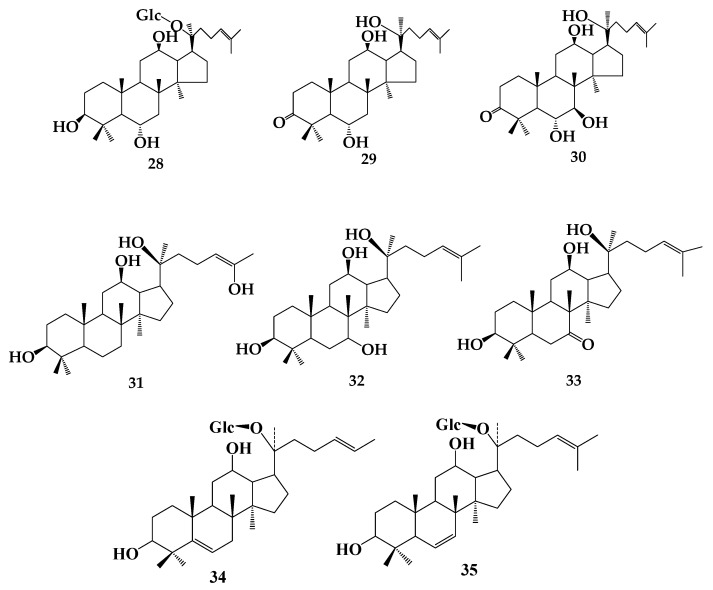
The chemical structures of compound **28**–**35**.

The biotransformation of ginsenosides is directed towards reducing glycosides. The rare ginsenosides with stronger pharmacological action can be obtained by biotransformation of ginsenosides, which have shown more unique and comprehensive efficacy in the treatment of tumor diseases and anti-inflammatory and bacteriostatic activity [23]. The **3′** metabolites **10**, **13**, and **14** have strong antiproliferation activity against lung cancer cell line A549. The metabolites of **8** cultured anaerobically with human fecal microorganisms showed strong cytotoxicity to tumor cell lines and could effectively inhibit the growth of *Helicobacter pylori* [24]. Liu et al. [25] found that *Absidia coerulea* AS 3.2462 could catalyze the dehydrogenation of ginsenoside **20** in C-3 and hydroxylation at 7β and 15α sites. The obtained metabolites ginsenoside F1 (**28**),3-oxo-20(S)-protopanaxatriol (**29**) and 3-oxo-7β-hydroxy-20(S)-protopanaxatriol (**30**) exhibited moderate reversal activity towards A549/taxol MDR tumor cells in vitro. More research showed that **14** and its transformed products, 26-hydroxy-20(S)-PPD (**31**), 7β-hydroxy-20(S)-PPD (**32**), and 7-oxo-20(S)-PPD (**33**), could inhibit the proliferation of DU-145 and PC-3 cancer cells [26]. The functional activities of ginsenosides are different due to their different structures, such as ginsenoside Rh1 and Rh2, which have antitumor activity, and **19** and **20**, which have anti-inflammatory and bacteriostatic effects. The application of ginsenosides has also made new progress in the activation and induction of osteogenic differentiation. Zhou et al. transformed **3** by *Paecilomyces bainier* sp. 229 to obtain four compounds: **10**, **12**, 5,6-ene-20-*O*-b-d-glucopyranosyl-20(S)-protopanaxadiol (**34**), 6,7-ene-20-*O*-b-d-glucopyranosyl-20(S)-protopanaxadiol (**35**). These compounds can osteogenic differentiation by activating the Wnt/β-catenin signaling pathway [27]. Therefore, the biotransformation of ginsenosides has been supported theoretically. (Figure 6).

## 3. Gypenoside

*Gynostemma pentaphyllum* (Thunb.) Makino is a *Gynostemma* plant of Cucurbitaceae, and is the only non-Araliaceae ginseng plant that contains ginsenosides. Gypenoside (Gyp) is the most important functional component of *Gynostemma pentaphyllum*, which has antiaging, anticancer, hypoglycemic, and lipid-lowering and neuroprotective effects. The aglycons of gypenoside and ginsenosides are all dammarane-type tetracyclic triterpenoids [28]. The absolute configurations of the chiral centers at C-20 are divided into R-configurations and S-configurations, while most of the glycosyl groups are connected to C-3 and C-20 sites, mainly including glucose, arabinose, rhamnose, and xylose. According to the similarity degree of structure, more than 160 types of gypenosides have been classified into 12 classes. There are 102 types of gypenosides with a dammar double bond at the C-24 and C-25 positions and 13 kinds of gypenosides have an epoxy structure at the C-21 and C-23 position of the side chain. In addition, the glycosylation of gypenosides by monosaccharides, disaccharides, or trisaccharides is mostly at C-3 and C-20, and consists of glucose, arabinose, rhamnose and xylose [29]. Among them, the structures of Gyp III, IV, VII, XII, I, and Gyp-A-AH were the same as those of ginsenoside **3**, **6**, **7**, **8**, **10**, and **25**, respectively. Interestingly, most of the secondary products hydrolyzed from gypenosides are the same as those hydrolyzed from ginsenoside, including **8** and **10**. In addition, the transformation among gypenosides can be achieved by changing the number of glycosyl groups [30]. The active ingredients in *Gynostemma pentaphyllum* are correlated with growth environment, species, and endogenous fungi. It has been reported that the *Fusarium* sp., column *Neurospora*, and *Leptosphaeria* can promote the synthesis and accumulation of gypenoside Gyp-XLIX, while *Penicillium* can inhibit its synthesis and accumulation [31].

**Figure 7:**
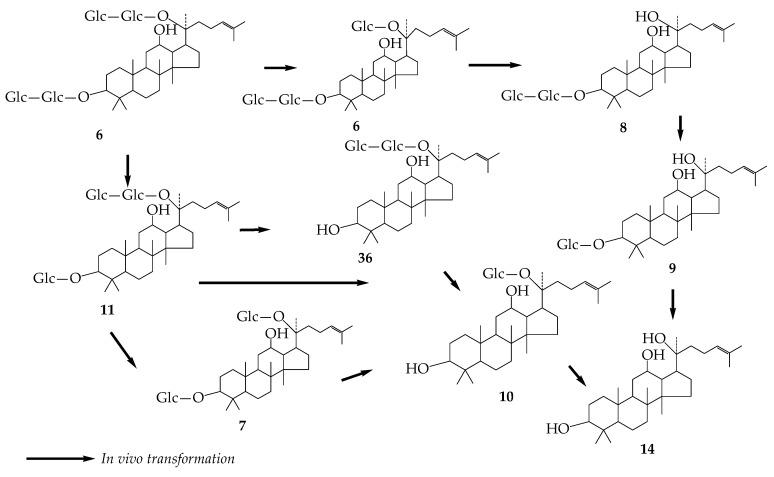
Metabolic pathway of Gyp-III in vivo.

At present, the microbial transformation of gypenosides is mainly based on ginsenosides, and the transformation pathway is similar to that of ginsenosides. However, the active ingredients of saponins need to be metabolized by intestinal flora to produce final products. Therefore, the intestinal flora is of great significance for the study of gypenosides. Six and eight metabolites were obtained by oral administration and intravenous injection of Gyp-LVI in rats, respectively [32]. Shen [33] obtained the main transformation pathway by anaerobic culture of Gyp-III with intestinal bacteria: 3→**11**→7/**26**→**10**. The main route is **11**→7 and the secondary route is **11**→Gyp-LXXV(**36**), but the final metabolite in both routes is **10**. A similar transformation pathway was obtained by *Leuconostoc mesenteroides* DC102 transformation of **6**. Therefore, the in vivo metabolic pathway of **6** can be roughly deduced (Figure 7) [34].

## 4. Glycyrrhizin

**Figure 8:**
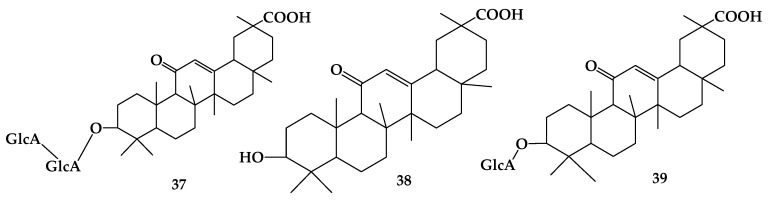
The chemical structures of compound **37**–**39**.

Glycyrrhiza is known as beet root or herbaceous plant and has anti-inflammatory activity, as well as functions in anti-allergic reaction, immunosuppression, and in the treatment of acute and chronic viral liver disease. Until now, 61 triterpenoids have been identified in *Glycyrrhiza*. Glycyrrhizic acid (GL, or named as glycyrrhizin (**37**)), which belongs to the pentacyclic triterpenoid saponins, is a component rich in *Glycyrrhiza*. Glycyrrhetinic acid (GA (**38**) is a hydrolytic product obtained from glycyrrhizin, and its chemical structure was determined in 1943 [35]. Due to its weak polarity as it lacks glycosyl, glycyrrhetinic acid is easily transported and absorbed in the blood, so its physiological activity is much stronger than that of glycyrrhizic acid. It is reported that glycyrrhetinic acid can inhibit the proliferation of murine melanoma B16 cells, while glycyrrhizin needs to increase the concentration by 20 times to achieve the corresponding effect [36]. Moreover, there is another substance between glycyrrhizic acid and glycyrrhetinic acid, glycyrrhetic acid monoglucuronide (GAMG (39)), which has better emulsifying and bubbling properties and stability than either glycyrrhizic or glycyrrhetinic acid (Figure 8). GAMG contains only one glucuronic acid group, showing moderate polarity and low lipophilicity, which confers better solubility and transmembrane transport ability in vivo [37]. The biotransformation of glycyrrhizin has various advantages including strong stereoselectivity and regioselectivity and low byproduct production as compared with the hydrolysis method. At the same time, it can improve the activity of active components and protect them from destruction.

**Figure 9:**
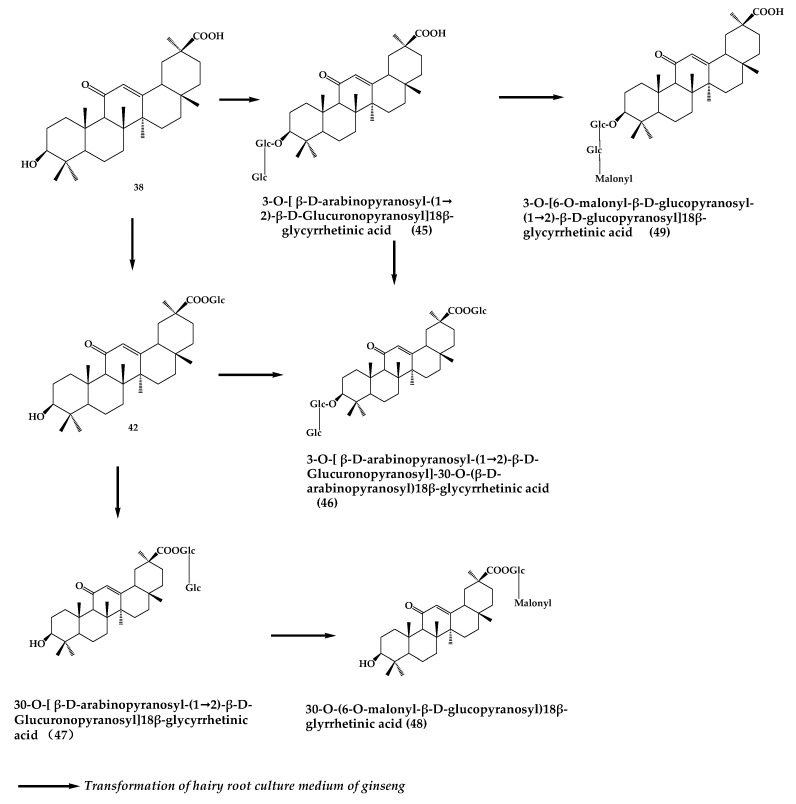
Glycyrrhetinic acid conversion pathway.

**Figure 10:**
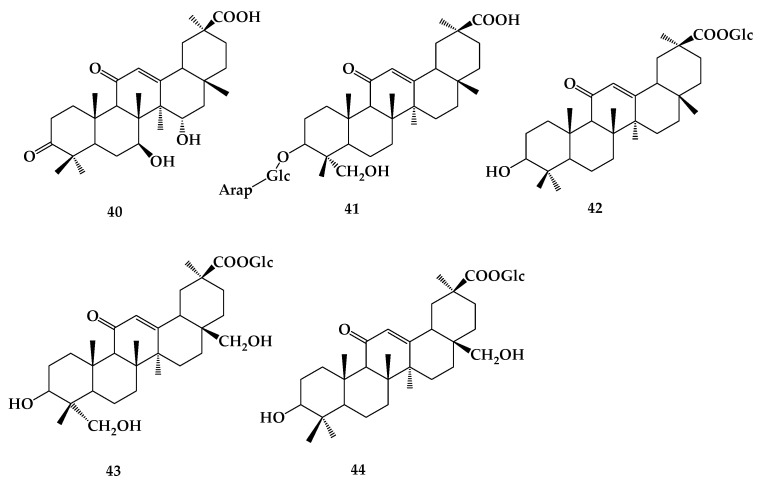
The chemical structures of compound **40**–**44**.

The biotransformation process of glycyrrhizin mainly comprises the steps of hydrolysis, hydroxylation, glycosylation, and acylation [38]. Glycyrrhizin can be converted into **39** or **38** by removing 1 or 2 molecules of glucuronic acid under the action of enteric bacteria or microorganisms. It was found that glycyrrhizin was converted to **39** together with a small amount of glycyrrhetinic acid by stepwise removal of glucuronic acid in lysosomes [39]. Hydroxylation of glycyrrhizin occurs mainly in the C–H bond of the triterpene ring, and *Aspergillus niger* can transform **38** to 7β,15α-diohydroxy-3,11-dioxo-oleana-12-en-30-oicacid (**40**) [40]. Glycosylation of glycyrrhizin is mainly achieved by biotransformation in plant suspension culture system, but not by microbial culture. It was found that the cell suspension culture of *Glycyrrhiza glabra* was able to convert **38** to new glucosides: 3-O-[α-l-arabinopyranosyl-(1→2)-β-D-glucuronopyranosyl]-24-hydroxy-18β-glycyrrhetinic acid (**41**) and 30-O-β-D-glycopyranosyl-18β-glycyrrhetinic acid (**42**) [41]. Six kinds of products were obtained after biotransformation of **38** using ginseng hairy root culture by the malonyl acylation of glucuronic acids (Figure 9) [42]. Moreover, studies have also shown that the use of free licorice suspension cells to transform licorice has the advantages of not affecting cell viability and physiological state, and the direct use of precursors. Orihara et al. [43] found that *Eucalyptus perriniana* cell culture can transform **38** into two products: 23,28-dihydroxy-18β-glycyrrhetinic acid-30β-glucopyranosyl ester (**43**) and 28-hydroxy-18β-glycyrrhetinic acid-30β-glucopyranosyl ester (**44**). Plant tissue and cell culture technology is an important way to obtain synthetic natural products, which has the advantages of short cycle and high repeatability. (Figure 10).

**Figure 11:**
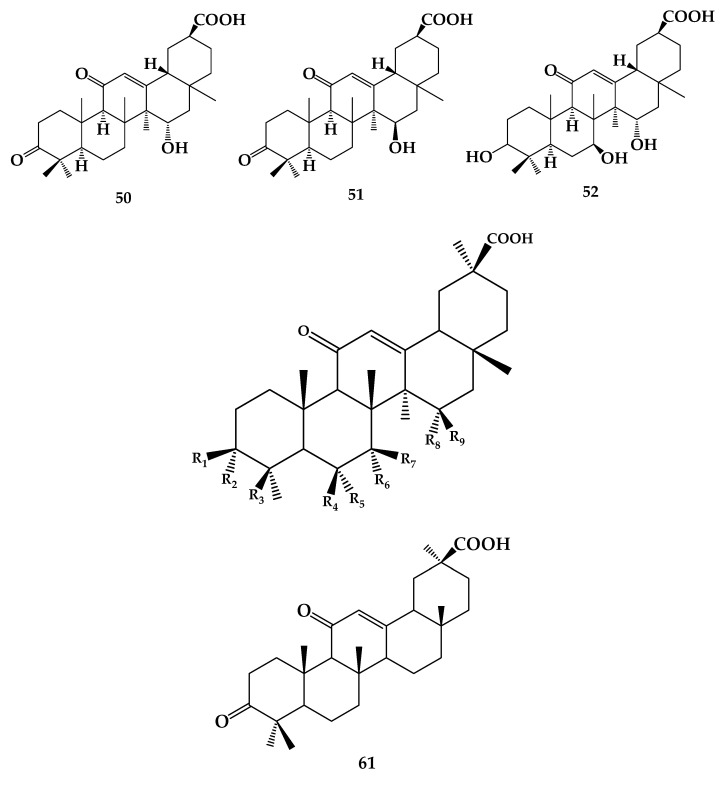
The chemical structures of compound **50**–**61**.

The essence of microbial transformation of glycyrrhizin is the catalytic effect of enzymes produced by microbial metabolism on the substrate. It has been reported that one major product and five secondary products were obtained after biotransformation of **38** by *Cunninghamella blakesleeana*. Their structures were identified as 3-oxo-15α-hydroxy-18β-glycyrrhetinic acid (**50**) (major product), 3-oxo-15β-hydroxy-18β-glycyrrhetinic acid (**51**), 7β,15α-dihydroxy-18β-glycyrrhetinic acid (**52**), **40**, 7β-hydroxy-18β-glycyrrhetinic acid (**53**), and 15α-hydroxy-18β-glycyrrhetinic acid (**54**) by MS, ^1^H NMR and ^13^C NMR analyses [44]. Eight metabolites were gained from 18β-GA after biotransformation using *Mucor polymorphosporus* (AS 3.3443), including **53, 54**, 24-hydroxyglycyrrhetinic acid (**55**), 6β-hydroxyglycyrrhetinic acid (**56**), 7α-hydroxyglycyrrhetinic acid (**57**), 3-O-acetyl-7β-hydroxyglycyrrhetinic acid (**58**), 3-oxo-7β-hydroxyglycyrrhetinic acid (**59**), 3-oxo-15α-hydroxyglycyrrhetinic acid (**60**) (Table 1) [45]. However, it was also reported that 18β-GA is the intermediate product of glycyrrhizic acid after biotransformation with *Aspergillus oryzae*, *Aspergillus niger*, and *Aspergillus sojae*, and the final product is 3-oxo-18β-GA (**61**) (Figure 11) [46]. It can be seen that different conversion products of active components in *Glycyrrhiza uralensis* were obtained after biotransformation using different microorganisms, and microbial transformation has selective hydroxylation, which needs further study.

Intestinal microorganisms are abundant in species, rich in enzymes, and mild in catalytic conditions which can catalyze reactions that are difficult to achieve by general chemical methods. Therefore, the study of intestinal microorganisms on the biotransformation of glycyrrhizin and related compounds is also of great significance. Akao [47] studied the differences of 18β-GL, 18β-GA, and 18β-GAMG in intestinal flora metabolism, and the results showed that 18β-GL could be transformed and metabolized by intestinal flora, but 18β-GL was not easily metabolized when coexisting with metabolites. The reason may be that metabolites, to some extent, affect the activity of β-D-glucuronidase produced by intestinal flora. It is also reported that glycyrrhizin and its secondary products can affect the absorption and metabolism of other drugs in the intestine. This conclusion was demonstrated by the experimental results of rats fed with different doses of glycyrrhizin and anticancer drug methotrexate [48]. In addition, Akao [49] found that the *Eubacterium* sp. GLH in the large intestine can produce two different enzymes, glycyrrhizin-β-D-glucuronidase and glycyrrhetic acid monoglucuronide-β-D-glucuronidase, which can hydrolyze glycyrrhizin and **39** to glycyrrhizic acid, respectively. According to the literature, there are two metabolic pathways of glycyrrhizin under the action of enterobacteria: the main pathway is the direct transformation of GL into **38**, and the secondary pathway is that **37** is transformed into **39** as an intermediate product and then transformed into **38** as the final product.

## 5. Saikosaponin

**Figure 12:**
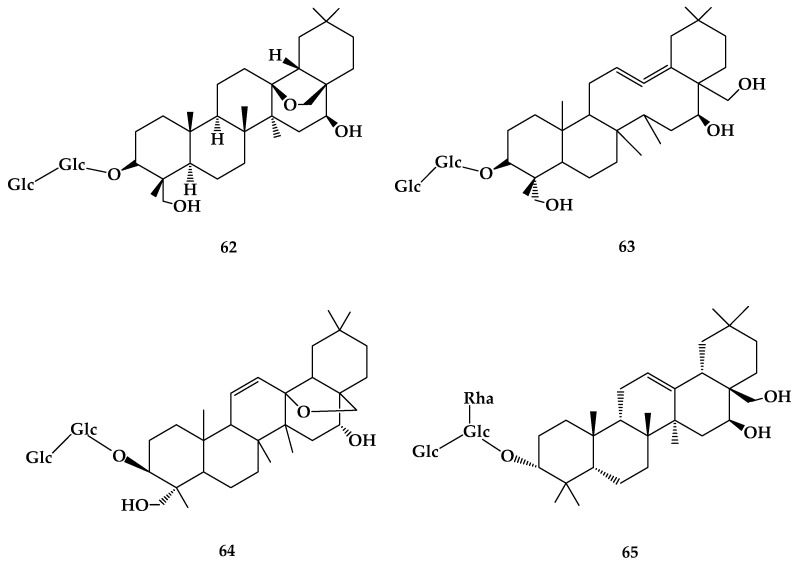
The chemical structures of compound **62**–**65**.

So far, about 200 species of *Bupleurum* are found worldwide [50]. Chai Hu has antiepileptic, antidepressant, anticancer, anti-inflammatory and immunomodulatory activities, anti-liver cancer and potential hepatotoxicity, and can relieve neuropathic pain [51]. There are over 100 kinds of saikosaponins, a pentacyclic triterpenoid oleanolic derivative, and they are the most important physiological active component of *Bupleurum*. Saikosaponins are found only in *Bupleurum* plants. Various saponins, such as saikosaponin a (**62**), saikosaponin d (**63**), saikosaponin c (**64**), and saikosaponin f (**65**), can be separated from Chai Hu, among which only saikosaponin a and d have demonstrated obvious pharmacological effects [52] (Figure 12).

Studies on the biotransformation of saikosaponins are mainly focused on the transformation in the gastrointestinal tract in vivo. The factors involved in the in vivo transformation of saikosaponins include intestinal flora, gastric acid, and enzymes, among which intestinal flora is the main research objective, including intestinal contents and single bacteria anaerobically isolated from feces. Shimizu [53] studied the transformation of saikosaponin in rat gastric juice and intestinal juice. Saikosaponin d was completely transformed into saikosaponin b2 within 30 min in the rat gastric juice environment, while saikosaponin a was almost completely transformed after 3 h. Seventy percent of saikosaponin a was transformed into saikosaponin b1 within the first 1 h, and the remaining saikosaponin a was transformed into saikosaponin g within 3 h. In a rat intestinal juice environment, saikosaponin a, b1, b2, d, and g were transformed into relative saikosaponin within 1 h with the same transformation rule. The transformation of saikosaponin c by human intestinal flora revealed four metabolites: prosaikogenin E1 (E1), prosaikogenin E2 (E2), prosaikogenin E3 (E3), and saikogenin E. It was also found that the intestinal bacteria of rats had similar metabolic pathways to saikosaponin c [54]. Moreover, *Bacteroides* JY-6 and *Bacteroides* YK-4, the bacteria isolated from human intestinal bacteria, could transform saikosaponin c to E via E1 (or E2) and E3. However, these bacteria were not able to directly transform E1 and E2 to saikogenin E. The hydrolytic transformation of saikosaponin b1 in normal rats, aseptic rats, and rats with specific intestinal microflora was studied. It was found that saikosaponin b1 could be hydrolyzed into corresponding prosaikogenin and saikosaponin under the intestinal microflora *Eubacterium* sp. A-44 and absorbed by intestinal tract [55]. Meselhy also found that the *Eubacterium* sp. A-44 isolated from human feces could hydrolyze and transform various saikosaponins, which further demonstrates the transformation and absorption of saikosaponin in intestinal tract [56].

## 6. Dioscin

Dioscin is a kind of active substance in Dioscoreaceae, Liliaceae, Caryophyllaceae, and Rosaceae plants, which has anticoagulant, anti-inflammatory, and antitumor effects, and reduces blood lipid. Dioscin is a kind of spirostanol saponin which is composed of diosgenin and a sugar chain on the C-3 hydroxyl group, and its hydrolysis product is diosgenin. Studies have shown that diosgenin is the main form exerting the pharmacodynamics of dioscin.

**Figure 13:**
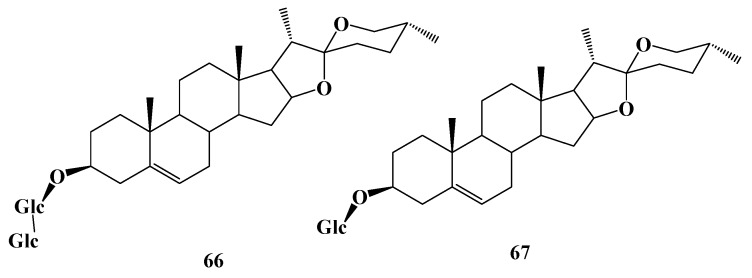
The chemical structures of compound **66,67**.

**Figure 14:**
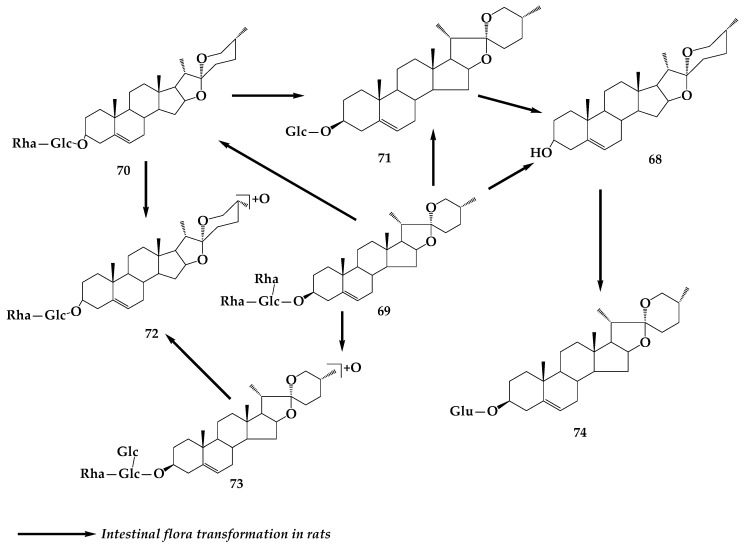
Transformation of diosgenin in rat intestinal flora.

**Figure 15:**
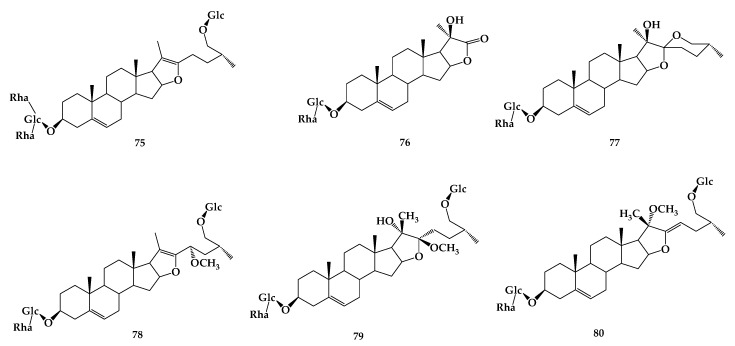
The chemical structures of compound **75**–**80**.

Gao et al. [57] transformed diosgenin by *Penicillium lilacinum* ACCC 31890. Three metabolites were isolated and identified as 5R-spirost-5-ene-3-ol-*O*-β-d-glucopyranoside-(1→3)-β-d-glucopyranosyl (**66**), trillin (**67**), (Figure 13) and diosgenin (**68**) with the conversion rate of 1%, 1%, and 45%, respectively. An in vitro study showed that the three products showed certain degrees of activity to inhibit the production of NO, IL-6, and MCP-1 in LPS-primed RAW264. 7 macrophages. Moreover, the anti-inflammatory activity of the bioconversion products increased along with the hydrolyzation of carbohydrate chain. **68** is the final product and shows the strongest anti-inflammatory activity among the three products. By studying the transformation of dioscin (**69**) in the intestinal flora of rats, it was found that two rhamnoses and one glucose group at the C-3 position were removed in turn and, thus, diosgenin A (**70**), diosgenin glucoside (**71**), and **68** were obtained. **69** and **70** can be oxidized to obtain two oxidation products e (**72**) and f (**73**). In the process of conversion, diosgenin can also be bound to form glucuronic acid glycosome. There are five pathways of transformation: **69**→**70**→**73**, **69**→**70**→**71**→**68**→g (**74**), **69**→**71**→**68**→**74**, **69**→**68**→**74**, **69**→**72**→**73** (Figure 14) [58]. Using a continuous process of enzymatic saccharification and microbial transformation, β-d-glucose or α-L-rhamnose was decomposed from the C-3 glucosidic bond of dioscin by *Trichoderma reesei*, and the yield of diosgenin was 90.6 ± 2.45% under optimum conditions [59]. More research has revealed that composite enzymatic hydrolysis is an effective pretreatment method for microbial hydrolysis after comparing the effects of physical separation, catalytic solvent extraction, ultrasonic fermentation, composite enzymatic hydrolysis, and enzymatic saccharification on the microbial transformation of dioscin [60]. Pseudoprotodioscin (**75**) can be transformed into five new oxidative metabolites by *Chaetomium olivaceum*, including 5-ene-3β,20β-diol-22,16-lactone-3-*O*-α-L-rhamnopyranosyl-(1→4)-β-d-glucopyranoside (**76**), (25R)-spirost-5-ene-3β,20β-diol-3-*O*-α-L-rhamnopyranosyl-(1→4)-β-d-glucopyranoside (**77**), 26-*O*-β-d-glucopyranosyl-23(S)-methoxyl-(25R)-furosta-5,20-diene-3β,26-diol-3-*O*-α-L-rhamnopyranosyl-(1→4)-β-d-glucopyranoside (**78**), 26-*O*-β-d-glucopyranosyl-22β-methoxy-(25R)-furosta-5-ene-3β,20β,26-triol-3-*O*-α-L-rhamnopyranosyl-(1→4)-β-d-glucopyranoside(**79**) and 26-*O*-β-d-glucopyranosyl-20α-methoxyl-(25R)-furosta-5,22-diene-3β,26-diol-3-*O*-α-L-rhamnopyranosyl-(1→4)-β-d-glucopyranoside (**80**) (Figure 15) [61].

## 7. Timosaponin

*Anemarrhena asphodeloides* Bunge is a plant that belongs to the family Liliaceae. Timosaponin is the most important chemical component in *Anemarrhena asphodeloides*, which has various physiological activities, such as anticancer, antidementia, antidepression, hypoglycemic, and lipid-lowering effects [62]. The saponins in *Anemarrhena asphodeloides* are abundant. According to different aglycone structures, they can be divided into spirosterol saponins (F ring is circular) and furostanol saponins (F ring is open chain). At present, timosaponin B-II (TB-II), timosaponin B-III (TB-III), and timosaponin A-III (TA-III) are the main research hotspots. To better understand the pharmaceutical mechanisms of TB-II, Yang et al. [63] prepared its deglycosylated derivatives through biotransformation with fungi *Colletotrichum gloeosporioides*, *Acremonium alternatum*, and *Aspergillus niger*. In the presence of 10g/L glucose, transformation with *C. gloeosporioides* yielded 4 products: TA-III and timosaponin AI (TA-I) and their β-isomers. TB-II is mainly hydrolyzed, dehydrated, and isomerized under microbial fermentation to produce a series of secondary saponins and corresponding isomers. Yao et al. [64] also found that TB-II can be hydrolyzed and isomerized by *C. gloeosporioides* to produce TA-III, TA-I, TA-III β-isomer, and TA-I β-isomer, while TA-III and TA-I were produced by *A. niger* transformation. When TA-III was co-cultured with *Saccharomyces cerevisiae* for 24 h, five metabolites were obtained, including TB-II, 5-hydroxy-TB-II, TB-III, 5-hydroxyTB-III, and a stereoisomer of TA-III with a specific isotype F-ring and β-ranged CH32–1 which rarely occurs in nature [65]. However, it has been reported that a potential industrial process for producing TA-III that involves biotransformation directly in the crude extract liquid of rhizoma anemarrhenae (RA) was developed. β-D-Glycosidase was used to transform TB-II into TA-III, and monofactor experiments were conducted to optimize the enzymolysis conditions. The best process condition was pH 4.0, 55 °C, 2 h, and the amount of β-D-glycosidase was 600 U/g [66].

**Figure 16:**
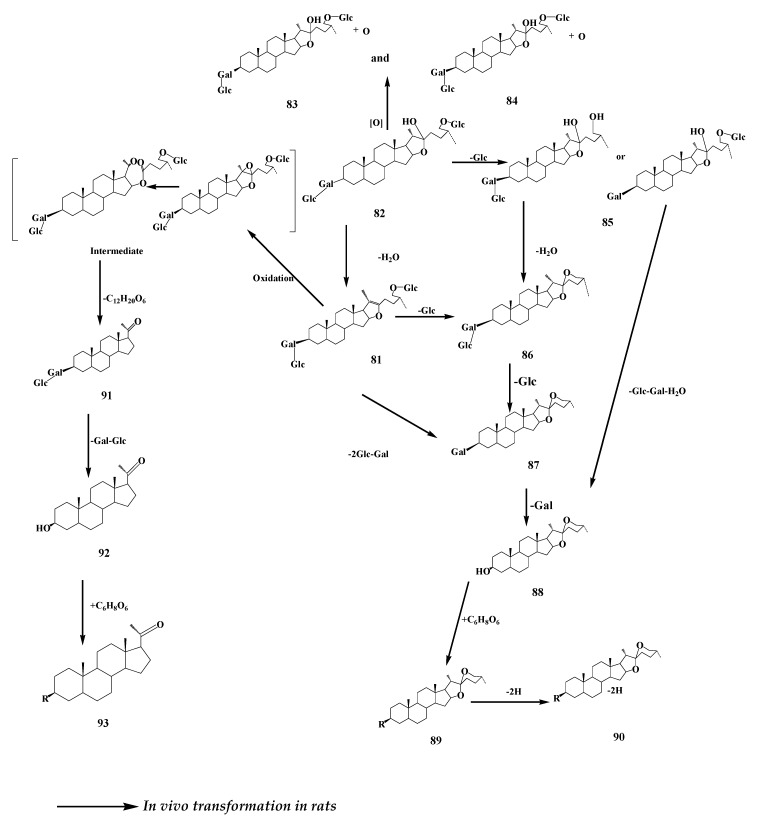
The proposed metabolic pathway of TB-II in rat [68].

Compared with biotransformation, timosaponins undergo more complex and diverse reactions under the action of intestinal flora, including deglycosylation, hydroxylation, and E-ring break rearrangement. Some studies have shown that TB-III (**81**) can be transformed into TB-II (**82**) and TA-III under the action of rat intestinal flora. It has been reported that TB-II monomer has been metabolized in rats and the final nine metabolites are TB-II, the hydroxylated metabolite of TB-II, two kinds of the hydroxylated metabolites of TB-III, deglycosylation and monooxygenation products of TB-III, the deglycosylation product of TB-II, TA-III, and the isomers of TA I-II [67]. A total of twelve metabolites were detected and identified by means of comparing molecular mass, retention time, and comparison of spectral patterns of the analytes with those of the parent drug. The products are monooxygenated B-II (**83**), monooxygenated B-II (**84**),**80**, deglycosylated B-II (**85**), timosaponin A-III^C^ (**86**), timosaponin A-I (**87**), sarsasapogenin^C^ (**88**), sarsasapogenin glucuronide (**89**), dehydrogenated sarsasapogenin glucuronide (**90**), glycosylated isopregnanolone (**91**), isopregnanolone^C^_2_ (**92**), isopregnanolone glucuronide (**93**). A possible metabolic pathway for the biotransformation of TB-II was also investigated and proposed [68] (Figure 16). It can be concluded that the main biotransformation methods include dehydration, single deglycosylation, double deglycosylation, hydroxylation, E-ring fracture, and oxidation reaction. In addition, because the stability of furosteric TB-II is weaker than that of spirosterane TA-III, the metabolites of TB-II are much more numerous than those of TA-III. Nine chemical constituents, magnoflorine, menisperine, palmatine, berberine, timosaponin N or timosaponin E1, timosaponin D, TB-III or anemarsaponin C or xilingsaponin B, TB-II, and TA-III, were detected by HPLC–DAD–MS in the serum of rats treated with timosaponin [69].

## 8. Astragaloside

*Astragalus membranaceus* belongs to the genus *Astragalus* of the butterfly flower family, and has the effects of enhancing immunity, protecting the liver, and antiaging effects. Results showed that the main active components of *Astragalus membranaceus* include saponins, polysaccharides, and flavonoids. Among saponins, astragaloside I (AS-I) (**94**), astragaloside II (AS-II) (**95**), astragaloside III (AS-III) (**96**), and astragaloside A (AS-IV) (**97**) are the four active substances with high contents [70]. Meng et al. studied the biotransformation characteristics of astragaloside components in human intestinal flora and obtained four products and determined their structures: **94**, **95**, **96**, and **97** [71]. AS-IV belongs to the lanolin alcohols shaped tetracyclic triterpene saponins, which has abundant clinical applications, but is almost nonexistent in the plant kingdom. The bioavailability of astragaloside is very low, so it is necessary to produce secondary saponins under the metabolism of intestinal flora, such as cycloastragenol, which has better permeability and absorptivity so that the bioavailability can be greatly improved [72]. Wang et al. [73] successfully transformed astragaloside IV (**98**) to cycloastragenol with *Bacillus* sp. by analyzing thin layer chromatography (TLC) and high-performance liquid chromatography (HPLC). Three metabolites were separated during the fermentation and characterized to be cyclogaleginoside B, cycloastragenol, and 20R,24S-epoxy-6α, 16β,25-trihydroxy-9,19-cycloartan-3-one based on NMR and MS spectroscopic analyses. Wei et al. [74] purified an acetyl esterase from *Absidia corymbifera* AS2, and this enzyme can hydrolyze the acetyl groups at positions O-2 or O-3 of the xylopyranosyl residue at the C-3 position of AS-I, isoAS-I, AS-II and isoAS-II, and convert these all to AS-I. The pathways of deacetylation catalyzed by this enzyme were clarified for the first time: AS-II→AS-IV, isoAS-II→AS-II→AS-IV, AS-I→ (AS-II, isoAS-II) →AS-IV and isoAS-I→AS-II→AS-IV.

**Figure 17:**
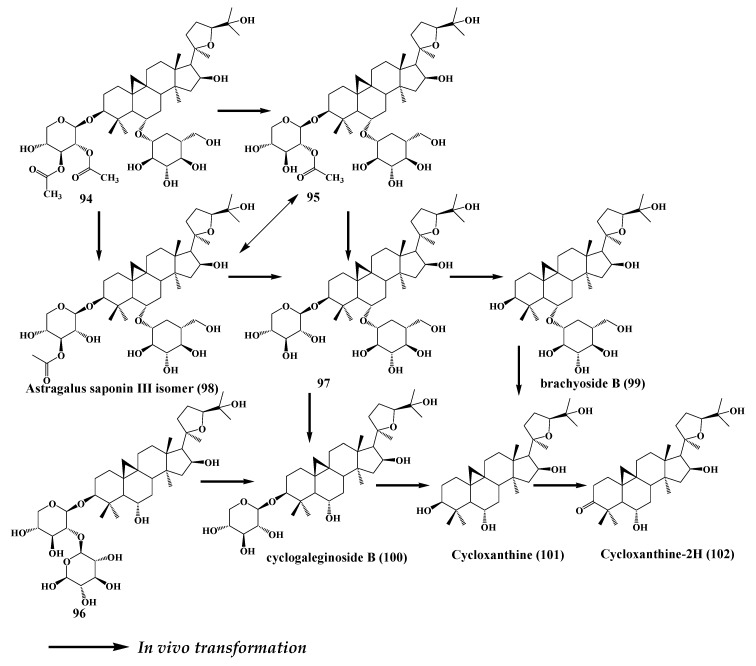
Astragaloside through human intestinal flora transformation pathway.

The main metabolic pathways of astragaloside in intestinal flora include deglycosyl, deacetylation, and dehydrogenation, and the common metabolite of astragaloside is cycloastragenol. The conversion efficiency of AS-I and AS-III was higher than that of AS-II and AS-IV. Astragaloside is gradually deglycosylated by human intestinal flora and transformed into cycloastragenol, which is the effective form in vivo. The specific transformation pathway is shown in Figure 17.

## 9. Ardipusilloside

**Figure 18:**
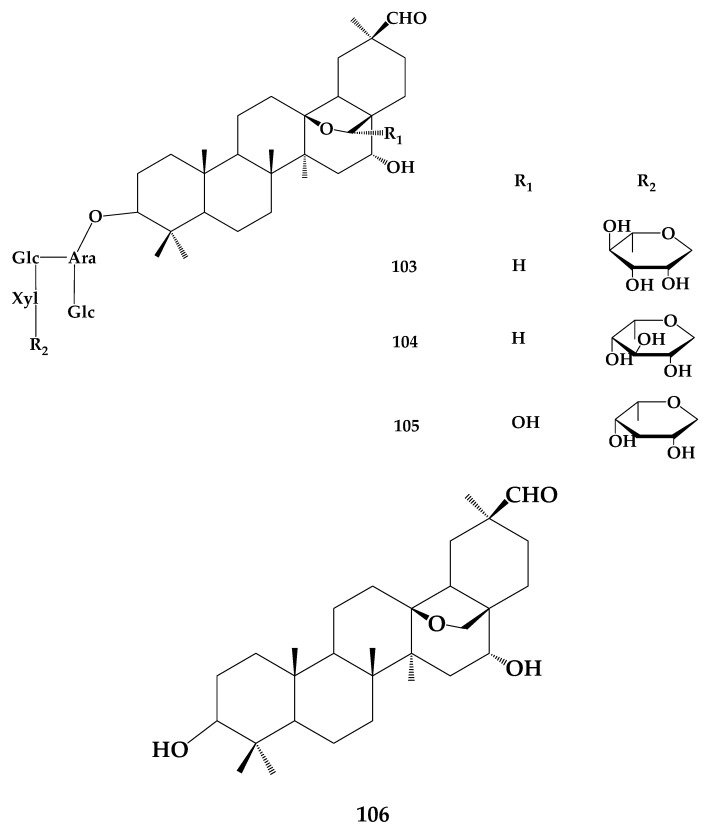
The chemical structures of compound **103**–**106**.

*Ardisia pusilla* A.DC. is a plant of the genus *Ardisia*, and contains the bioactive component ardipusilloside. Ye et al. [75] systematically classified ardipusillosides into ADS-I (**103**), ADS-II (**104**), ADS-III (**105**), and ADS-IV (**106**) (Figure 18). Due to its high anticancer activity, the triterpenoid saponin ADS- I has become the main research objective. However, **103** itself is not readily absorbed and needs to be metabolized to the deglycosylated form of ADS-I in order to achieve a better antitumor effect.

**Figure 19:**
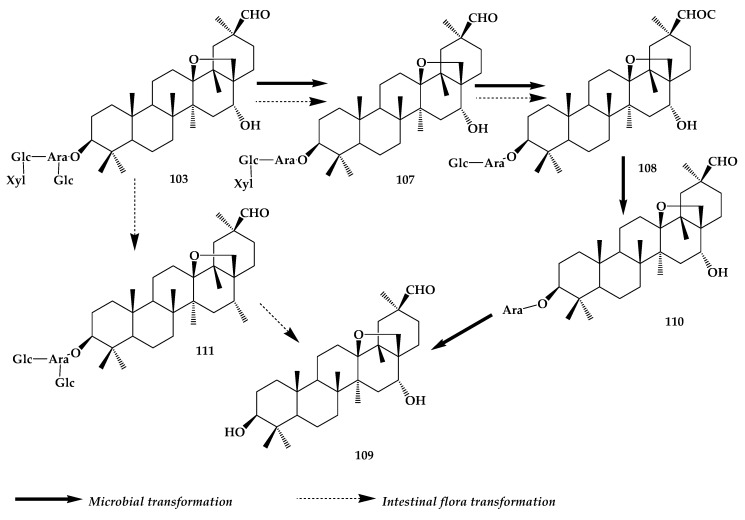
Transformation pathway of ADS-I.

**99** can produce the corresponding secondary saponins by deglycosylation of intestinal flora. Deglycosylation occurs mainly on the C-3 position of the sugar chain, and five secondary saponins can be obtained by removing the various glycosyl groups in turn. Comparing the biotransformation products of human intestinal flora with rat intestinal flora, three common secondary metabolites can be obtained: 3-O-[α-L-rhamnose(1→2)-β-D-glucopyranose(1→3)]-α-L-arabinose-cyclamiretin A (**107**), 3-O-[β-D-glucopyranose(1→3)]-α-L-arabinose-cyclamiretin A (**108**), aglycone of the ADS-I (**109**) [76] (Figure 19). However, there are obvious differences in the quantity and species of saccharides removed under the action of human intestinal flora and rat intestinal flora. Under the transformation of rat intestinal flora, **103** can specifically hydrolyze rhamnosides to produce the secondary saponin cyclamiretin-A-β-d-cyclamiretin-(1→2)-β-d-glucopyranosyl group-(1→3)- α-L-arabinopyranoside (**110**). In the human gut, **103** can specifically remove one rhamnose and two glucosyl groups to obtain the secondary saponin cyclamiretin-A-3-β-d-arabinopyranoside (**111**). At the same time, they also studied the culture of human fresh fecal extract and saponins in anaerobic conditions at 37 °C. Through deglycosylation, two sub-glycosides (A, B) and one glycoside (C) were finally produced. ADS-I and its metabolites have a good inhibitory effect on the growth of cancer cells [76,77].

## 10. Outlook

The wide chemical diversity of both triterpenoid and steroidal saponins has resulted in renewed interest and investigations of these compounds in recent years. Unlike other natural products, such as alkaloids, the bioavailability of original saponins after oral administration is relatively low, but rare saponins with relatively high bioavailability can be obtained by biotransformation. Biotransformation requires the structural modification of saponins to efficiently and steadily obtain the target compounds, for example, through the synergy of various microorganisms. By using multiple strains with the same activity, insoluble compounds such as saponins can be synergistically transformed. In the study of metabolism in vivo, saponins can be transformed to different compounds in the gastrointestinal tract, mainly mediated by intestinal flora. Intestinal flora has a strong metabolic function on saponins, and the main metabolic mode is the step-by-step hydrolysis of sugar. The metabolism of intestinal flora is an important link to improve the bioavailability of saponins and enhance their pharmacological activity. The biotransformation pathways of natural saponins have been gradually discovered, which provides an effective scientific basis for the study of saponin metabolism and synthesis.

## Figures and Tables

**Table 1:** The chemical structures of compound **53**–**60**.

	R1	R2	R3	R4	R5	R6	R7	R8	R9
**53**	**OH**	**H**	**CH_3_**	**H**	**H**	**H**	**OH**	**H**	**H**
**54**	**OH**	**H**	**CH_3_**	**H**	**H**	**H**	**H**	**OH**	**H**
**55**	**OH**	**H**	**CH_2_OH**	**H**	**H**	**H**	**H**	**H**	**H**
**56**	**OH**	**H**	**CH_3_**	**OH**	**H**	**H**	**H**	**H**	**H**
**57**	**OH**	**H**	**CH_3_**	**H**	**H**	**OH**	**H**	**H**	**H**
**58**	**OAc**	**H**	**CH_3_**	**H**	**H**	**H**	**OH**	**H**	**H**
**59**	**O**	**O**	**CH_3_**	**H**	**H**	**H**	**OH**	**H**	**H**
**60**	**O**	**O**	**CH_3_**	**H**	**H**	**H**	**H**	**OH**	**H**
